# Intraoperative laser angiography using the SPY system: review of the literature and recommendations for use

**DOI:** 10.1186/1750-1164-7-1

**Published:** 2013-01-07

**Authors:** Geoffrey C Gurtner, Glyn E Jones, Peter C Neligan, Martin I Newman, Brett T Phillips, Justin M Sacks, Michael R Zenn

**Affiliations:** 1 Stanford Hospital and Clinics, Palo Alto, CA, USA; 2 Peoria Surgical Group, Peoria, IL, USA; 3 University of Washington Medical Center, Seattle, WA, USA; 4 Cleveland Clinic Florida, Westin, FL, USA; 5 Stony Brook Medical Center, Stony Brook, NY, USA; 6 Johns Hopkins School of Medicine, Baltimore, MD, USA; 7 Plastic and Reconstructive Surgery, Duke University Medical Center, Durham, NC, USA

**Keywords:** SPY, Indocyanine green, Laser angiography, Reconstructive surgery, Perfusion assessment, Flap, Near infrared, Necrosis, Flap, Microsurgery

## Abstract

Inadequate tissue perfusion is a key contributor to early complications following reconstructive procedures. Accurate and reliable intraoperative evaluation of tissue perfusion is critical to reduce complications and improve clinical outcomes. Clinical judgment is the most commonly used method for evaluating blood supply, but when used alone, is not always completely reliable. A variety of other methodologies have been evaluated, including Doppler devices, tissue oximetry, and fluorescein, among others. However, none have achieved widespread acceptance. Recently, intraoperative laser angiography using indocyanine green was introduced to reconstructive surgery. This vascular imaging technology provides real-time assessment of tissue perfusion that correlates with clinical outcomes and can be used to guide surgical decision making. Although this technology has been used for decades in other areas, surgeons may not be aware of its utility for perfusion assessment in reconstructive surgery. A group of experts with extensive experience with intraoperative laser angiography convened to identify key issues in perfusion assessment, review available methodologies, and produce initial recommendations for the use of this technology in reconstructive procedures.

## Introduction

One of the fundamental causes of early complications following reconstructive procedures is considered to be inadequate tissue perfusion [1-6]. Therefore, accurate and reliable intraoperative evaluation of tissue perfusion is critical to reduce complications and improve clinical outcomes. Clinical judgment is the most widely used method for evaluating blood supply [7], but by itself is not always completely reliable for assessment of flap perfusion [8,9]. Several technologies to assess tissue vascularity have been evaluated in studies and used clinically, but none have achieved universal acceptance [6,10].

Intraoperative laser angiography using indocyanine green (ICG) is a vascular imaging methodology that can be used in the intraoperative or postoperative setting to visually assess superficial blood flow. Characteristics of ICG angiography make it a desirable option for tissue evaluation. The technique provides real-time assessment of tissue perfusion that has been correlated with clinical outcomes [1,5,6,11,12] and guides surgical decision making, such as flap design or intraoperative tissue resection [13]. ICG has an excellent safety profile and short plasma half-life, allowing for repeat evaluations during the same operative procedure. Although this technology has been used for decades in ophthalmology, its introduction to reconstructive surgery is relatively recent, and surgeons may not be aware of its characteristics, indications in reconstructive surgeries, and best practices in the intraoperative setting.

In May of 2011, a group of 7 experts (6 plastic surgeons and 1 general surgery resident) with extensive experience in the use of SPY Intraoperative Perfusion Assessment System (distributed in North America by LifeCell Corp., Branchburg, NJ; manufactured by Novadaq Technologies Inc., Richmond, British Columbia, Canada) convened a summit to develop a review describing the current state of the art of evaluation of tissue perfusion in reconstructive surgery. All of the authors use the SPY System, which has received FDA clearance for use in cardiovascular procedures; plastic, micro- and reconstructive surgical procedures; organ transplant; and gastrointestinal surgical procedures. The goals of this summit were to describe clinical uses for the SPY System in reconstructive surgical procedures and to produce a set of technical recommendations for its use. An extensive literature review was conducted to identify relevant studies, and the recommendations described in this review are based on the best available evidence.

## Background

Dissection of tissue of any kind is accompanied by some degree of devascularization, and accurate determination of tissue viability is critical to all surgeons. Loss of skin flaps due to ischemia and postoperative necrosis can be catastrophic and may result in an even larger tissue deficit requiring repair. Early identification of insufficiently perfused tissue with the potential to develop ischemia or postoperative necrosis will help guide intraoperative decision making, such as the need for flap revision, tissue resection, or a delayed procedure.

The wide variation in anatomic location of perforating vessels and their perfusion zones makes vessel selection and flap design a clinical challenge. Therefore, a reliable method to identify perforating vessels and their perfusion zones, assess tissue perfusion, and identify tissue at risk for necrosis would be valuable to surgeons. Clinical judgment alone remains the most commonly used intraoperative method to determine the degree of tissue perfusion [7]. Although clinical judgment is an important tool and provides relevant information, studies have demonstrated that clinical assessment alone can at times be a poor predictor of postoperative complications [8,9,12].

The SPY System provides a clinically useful assessment of perfusion in a variety of applications, including free and pedicle flaps and mastectomy and other skin flaps [14]. The technique allows for visualization of arterial inflow, venous return, and tissue perfusion during the intraoperative period. The information provided by this technique augments and strengthens clinical judgment and provides the surgeon with real-time visualization of tissue perfusion and vascular changes that result from surgical maneuvers.

The SPY System utilizes a fluorescence agent, indocyanine green (ICG), to enable visualization, similar to fluorescein in the past. Certain characteristics of ICG make it a more beneficial tool for intraoperative perfusion assessment. The ICG molecule binds strongly to plasma proteins, causing it to remain in the intravascular space. It also has a short plasma half-life of 3 to 5 minutes in humans [15]. This pharmacokinetic profile allows for rapid clearance of dye from tissues and repeated evaluations during the same surgical procedure. In comparison, the dye fluorescein stays in the tissue for more than 12 hours, meaning that it can be used only once. ICG is administered by means of peripheral or central intravenous access, is excreted exclusively by the liver into the bile, and is not associated with risk for nephrotoxicity. The laser diode array utilized by the SPY System emits a near-infrared wavelength that does not require the use of protective eyewear or other safety equipment.

The SPY System is currently indicated for capturing and viewing fluorescence images for the visual assessment of blood flow as an adjunctive method for the evaluation of tissue perfusion and related tissue-transfer circulation in tissue and free flaps used in plastic, micro-, and reconstructive and gastrointestinal surgical procedures. The system is used in evaluating recipient site vascularity as well as circulation in tissue flaps and free flaps used in plastic, microsurgical, and reconstructive surgical procedures [15]. For plastic and reconstructive surgery applications, the SPY System can be used to evaluate perfusion in all components of the flap, including skin, fat, fascia, muscle, periosteum, nipple (in breast reconstruction, reduction, and mastopexy), and arterial and venous flow in vessels (in microsurgery).

The recommendations and the authors’ personal experiences pertain to the SPY Intraoperative Perfusion Assessment System. This system utilizes a laser diode array to illuminate a maximum field of 18.5 × 13.5 cm^2^[15]. A charge-coupled device camera can be configured to capture image sequences at 3.75 to 30 frames per second, depending on the desired recording time of between 30 seconds and up to a maximum of 4.5 minutes. Images are viewed on a high-definition monitor in real time, allowing for immediate evaluation in the OR. The system includes computer hardware and software for capturing, enhancing visualization, and archiving of images and creation and printing of imaging procedure reports [11]. A companion post-processing software product (SPY-Q™ Analysis Toolkit) provides additional viewing, comparison, and analysis tools, including algorithms for measurement of fluorescence intensity. The clinician retains ultimate responsibility for making the pertinent diagnosis based on their standard clinical practices and visual comparison of the images.

### Modalities for assessment of tissue perfusion

Clinical judgment for flap evaluation involves subjective indices, such as tissue color, capillary refill, flap temperature, and dermal bleeding. When used alone, even by experienced surgeons, clinical judgment may inaccurately assess adequacy of tissue perfusion and predict ultimate outcomes.

Surgeons have evaluated and incorporated adjunctive modalities for assessment of vascular anatomy and, in some cases, tissue perfusion. Multiple modalities have been used clinically and described in the literature, including various Doppler devices, computed tomography angiography (CTA), magnetic resonance angiography (MRA), tissue oximetry, and fluorescein, among others (Table 1) [1,8,9,11,12,14,16-52].

**Table 1:** Methods for evaluation of tissue perfusion, by setting

**Method/Setting**	**use**	**Advantages**	**Limitations**	**Sources**
**Intraoperative**				
ICG intraoperative laser angiography*	→Visualize perforator perfusion zone in real time	→Visualizes perforator perfusion zones	→Requires administration of contrast media	Phillips et al., 2012 [12]
	→Confirm patency of arterial and venous anastomoses	→No exposure to ionizing radiation	→Does not identify precise vessel location or course through muscle and fascia when lipodystrophy exists	Francisco et al., 2010 [52]
	→Confirm perfusion of tissue prior to incision, after elevation of flaps, and prior to final closure	→Strong safety profile and short half-life of ICG		Komorowska-Timek & Gurtner, 2010 [1]
		→Permits re-evaluation during same surgery		Murray et al., 2010 [16]
				Tamburrino et al., 2010 [17]
				Newman et al., 2009 [11]
				Jones et al., 2009 [14]
				Azuma et al., 2008 [18]
				Prantl et al., 2008 [19]
				De Lorenzi et al., 2005 [20]
				Mothes et al., 2004 [8]
				Holm, Tegeler, et al., 2002 [21]
				Holm, Mayr, et al., 2002 [22]
				Still et al. 1999 [23]
Doppler – handheld	→Identification of perforator vessel location	→Easy to use	→Provides information on discrete area below probe	Yu & Youssef, 2006 [29]
		→Widely available	→Requires direct skin contact	
		→Inexpensive	→Does not identify perforator perfusion zone	
		→Provides confirmatory information	→Provides limited data and accuracy for flap design,	
			→especially in heavier patients	
			→Difficult to quantify	
			→Does not stratify perforators	
Fluorescein	→Visualization of perforator perfusion zone	→Visualization of perforator perfusion zone	→Single use only	Phillips et al., 2012 [12]
		→Widely available	→No venous information	Losken et al., 2008 [51]
			→Long delay time	
			→Toxicity concerns	
			→Use of ultraviolet Woods lamp	
			→High sensitivity, low specificity	
**Preoperative**				
Clinical judgment	→Estimation of tissue perfusion and flap viability	→Familiarity, ease of use	→Poor reliability when used alone	Phillips et al., 2012 [12]
			→Dependent on surgeon experience	Mothes et al., 2004 [8]
			→Inferior to imaging modalities for estimation of flap survival	Olivier et al., 2003 [9]
				Holm, Tegeler et al., 2002 [21]
Doppler Ultrasound (duplex, color, power)	→Identification of perforator vessel location	→No exposure to ionizing radiation or contrast media	→Inferior to CT angiography for identification of vessel location	Rozen et al., 2008 [24]
	→Estimate of vessel flow rate	→Provides estimation of perforator location, caliber, and flow	→Considered operator-dependent	Khalid et al., 2006 [25]
			→Does not identify perforator perfusion zone	Giunta et al., 2000 [26]
			→High rate of false-positive findings reported	Hallock, 2003 [27]
				Blondeel et al., 1998 [28]
Laser Doppler flowmetry	→Identification of vessel location and tissue perfusion	→No exposure to ionizing radiation or contrast media	→May underestimate flap survival	Schlosser et al., 2010 [30]
		→Identifies ischemia in flaps	→Poor ability to detect perforator vessels	Holzle et al., 2006 [31]
			→Sensitive to small movements	Heller et al., 2001 [32]
				Heden et al.1986 [33]
CT angiography	→Visualization of location and course of vessels through muscles and fascia	→Accurate detection of anatomic location and course of vessels	→Does not assess vascular flow	Ghattaura et al.,2010 [34]
		→Greater accuracy than Doppler ultrasound	→Does not show perforator perfusion zones	Smit et al., 2009 [35]
		→Potential for reduced surgical time	→May have poor resolution for vessel caliber;	Rozen et al., 2008 [24]
			→Exposure to ionizing radiation	Cina et al., 2010 [36]
			→Potential toxicity of contrast media	Scott et al., 2010 [37]
				Phillips et al., 2008 [38]
				Rosson et al., 2007 [39]
				Masia et al., 2006 [40]
MR angiography	→Visualization of location and course of vessels through muscles and fascia	→Greater accuracy than Doppler ultrasound	→Does not assess vascular flow	Schaverien et al., 2011 [42]
		→Detection of small caliber vessels	→Does not show perforator perfusion zones	Newman et al., 2010 [43]
		→Potential for reduced surgical time	→Less spatial resolution compared to CT angiography	Greenspun et al., 2010 [45]
		→No exposure to ionizing	→Potential toxicity of contrast	Chernyak et al., 2009 [41]
		→radiation	→media	Neil-Dwyer et al., 2009 [44]
				Rozen et al., 2009 [46]
**Postoperative**				
Transcutaneous oxygen monitoring**	→Assessment of tissue oxygen saturation	→Useful for postoperative monitoring	→Limited to discrete 1 cm^2^ area under probe	Steele, 2011 [47]
		→Accurately detects vascular compromise	→Numeric output only	Lin et al., 2011 [48]
		→Improves flap salvage rate in postoperative setting	→Used primarily for postoperative monitoring	Keller, 2009 [49]
			→Time consuming, cumbersome for intraoperative mapping	Keller, 2007 [50]

* Includes evidence from use of ICG intraoperative perfusion assessment devices available outside the United States.

** Used intraoperatively by some surgeons.

*CT*: computed tomography.

*MR*: magnetic resonance.

*ICG*: indocyanine green.

Intraoperative use of hand-held Doppler is safe, simple, and widely available. Several authors have described the preoperative use of Doppler ultrasound to identify vessel location [27,28]. However, the technique is considered an operator-dependent procedure [27], does not identify the perforator perfusion zone, and provides limited information and accuracy for flap design [29]. Evidence suggests that Doppler ultrasound may be reliable for estimation of vessel caliber.

It has been suggested that laser-Doppler flowmetry can be used for evaluation of blood flow within flaps. However, when used intraoperatively, it may underestimate flap survival [33]. The technique is also sensitive to small movements and is cumbersome. A recent study reported that although laser Doppler was effective at identifying ischemia in free flaps, it had poor ability to detect perforator vessels [30].

CTA and MRA are often used for preoperative evaluation of perforator vessel location. These methods provide accurate visualization of the anatomic course of vessels through the muscle and fascia [24,34-46]. However, neither provides information regarding blood flow through these vessels, nor the extent of perfusion from the perforator. Information provided by CTA/MRA and intraoperative laser angiography with ICG can be considered complementary; CTA and MRA accurately identify vessel location, whereas ICG provides information regarding the real-time location and extent of perfusion at the level of the skin.

Transcutaneous oxygen monitoring has been shown to improve flap salvage rates when used for postoperative monitoring [47,48]. Some investigators have brought this technique into the intraoperative setting, but it can be time consuming and labor intensive for mapping flaps. Initial findings suggest that it may have utility for continuous monitoring of flap perfusion [49,50].

Fluorescein angiography has been used to evaluate tissue perfusion in the operating room. The limitations of fluorescein include a long half-life, rapid leakage from capillaries into the interstitium, and a clearance time of 12 to 18 hours, which precludes re-evaluation during the intraoperative period [22,53-55]. Local ischemia enhances fluorescein diffusion, potentially leading to false-positive results [53,56]. Recent data suggest that fluorescein is not as accurate as intraoperative laser angiography, with lower specificity and negative predictive value [12,21,22,51]. Toxicity of the contrast medium is also a concern.

### Evidence of utility for intraoperative Use of the SPY system

ICG has been successfully used to evaluate perfusion in ophthalmologic procedures for decades [57,58]. More recently, the technology was adopted for use in cardiac [59,60], vascular [61,62], and transplant surgery [63]. Over the last decade, surgeons have applied the technology to plastic reconstructive surgical procedures, demonstrating clinical utility [1,11,12,16,22,52].

#### Use of SPY intraoperative perfusion assessment system in reconstructive surgeries

Clinical experience with intraoperative laser angiography for visualization of tissue perfusion during reconstructive surgical procedures is summarized in Table 2. Studies in reconstructive surgical procedures have reported good correlation between ICG-visualized circulation and postoperative outcomes [13,23,64-66]. In case series and prospective studies, adequate intraoperative perfusion assessment using SPY was associated with reduced rates of postoperative necrosis and flap loss compared to clinical judgment alone in a variety of reconstructive procedures, including free flap, pedicle flap, and implant reconstructions [1,5,8,11,14,18,20-22,67].

**Table 2:** Clinical evidence of utility the SPY system, by application

**Authors**	**Study design**	**Applications studied**	**Key points**
**Free flaps**			
Pestana et al., 2009 [5]	Case series	Multiple indications: head and neck, breast, lower extremity	1 partial flap loss
	N = 27 patients, 29 free tissue transfers		
**Breast recon-struction**			
Phillips et al., 2012 [12]	Prospective study comparing ICG to fluorescein and clinical judgment	Tissue expander-implant breast reconstruction	ICG and fluorescein had sensitivity of 90% and specificity of 50% and 30%, respectively; negative predictive value for ICG and fluorescein was 88% and 82%, respectively.
	N = 32 patients, 51 breasts		
Newman et al., 2011 [6]	Case series	Breast reconstruction: single-pedicle TRAM	ICG perfusion assessment identified perfusion zones; no issues with wound healing or tissue or fat necrosis.
	N = 20		
Komorowska-Timek and Gurtner, 2010 [1]	Case series	Breast reconstruction: tissue expander, latissimus dorsi flaps, DIEP/SIEA	Tissue expander (n = 16), latissimus dorsi (n = 2), DIEP/SIEA (n = 6); complication rate: 4% with ICG vs. 15.1% in 206 previous reconstructions (n = 148; *p* < 0.01)
	N = 20 patients, 24 breasts		
Tamburrino et al., 2010 [17]	Retrospective analysis	Breast reconstruction: tissue expander or unilateral TRAM	95% correlation between ICG imaging and clinical outcome, 100% sensitivity and 91% specificity.
	Tissue expander (n = 11 patients, 19 breasts)		
	Unilateral TRAM (n = 1)		
Francisco et al., 2010 [52]	Case series N = 5	Breast reconstruction: DIEP	No flap loss, fat necrosis, or take-backs
Jones et al., 2009 [14]	Case series	Breast reconstruction: free and pedicle TRAM, DIEP, latissimus dorsi, and expander insertions.	Of 5 patients with poor flap perfusion on ICG imaging, 4 developed necrosis and 1 blistering in a pattern predicted by ICG; necrosis rate of 6.3% vs. published rates of 15-25%.
	N = 43 patients, 64 breasts		
Newman & Samson, 2009 [11]	Case series	Breast reconstruction: DIEP or free TRAM	ICG detected marginal or poor perfusion in 4 cases; 3 were revised intraoperatively and the 1 that was not revised required return to OR for venous congestion. Flap survival was 100%.
	N = 8 patients, 10 breasts		
**NAC evaluation**			
Murray et al., 2010 [16]	Case series	Breast reduction surgery	ICG used to demonstrate NAC perfusion and venous outflow during surgery.
	N = 12 patients, 22 breasts		

*DIEP*: deep inferior epigastric perforator.

*NAC*: nipple-areolar complex.

*SIEA*: superficial inferior epigastric artery flap.

*TRAM*: transverse abdominus musculocutaneous.

### Recommendations for the intraoperative Use of SPY

**Figure 1:**
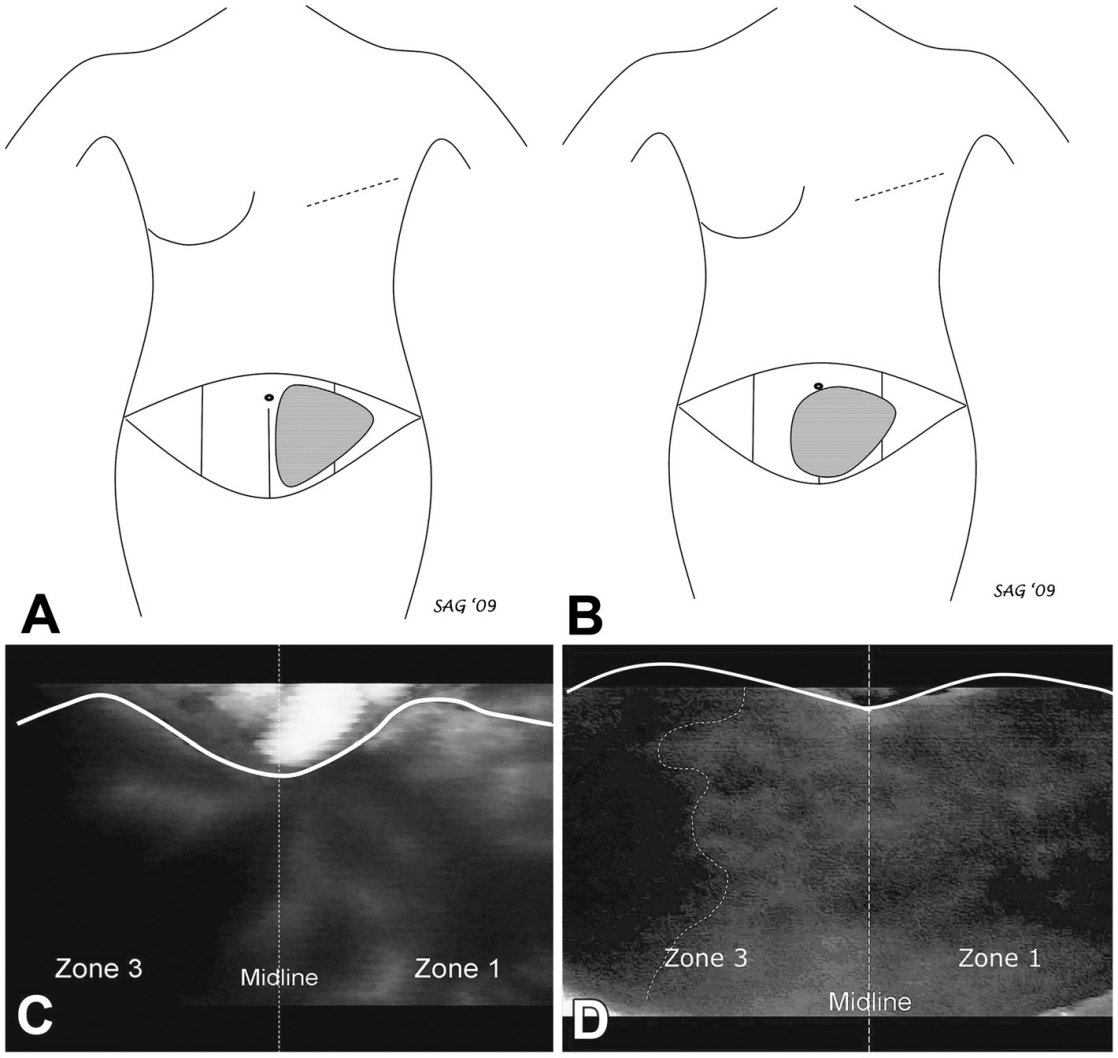
**Illustration of the use of SPY to assist in the design of transverse abdominus musculocutaneous (TRAM) flap for breast reconstruction in two patient cases.** Panel **A **shows a schematic illustration of the best perfused tissue in the TRAM flap for Case 1. This flap was designed based on the corresponding SPY image (Panel **C**), which demonstrated good perfusion in zone 1, clearly definable and in contrast to the poor perfusion across the midline in zone 3. In contrast, Panel **B **shows the schematic flap design for the TRAM flap in Case 2 based on the SPY image (Panel **D**), which demonstrated good perfusion in zone 1 and to a clearly definable point in zone 3 (dotted line). Lateral to this point in zone 3, fluorescence on SPY remained poor, indicating areas of relative ischemia and potential tissue loss. This information was originally published in Can J Plast Surg 2011;19(1):e1-e5.

**Figure 2:**
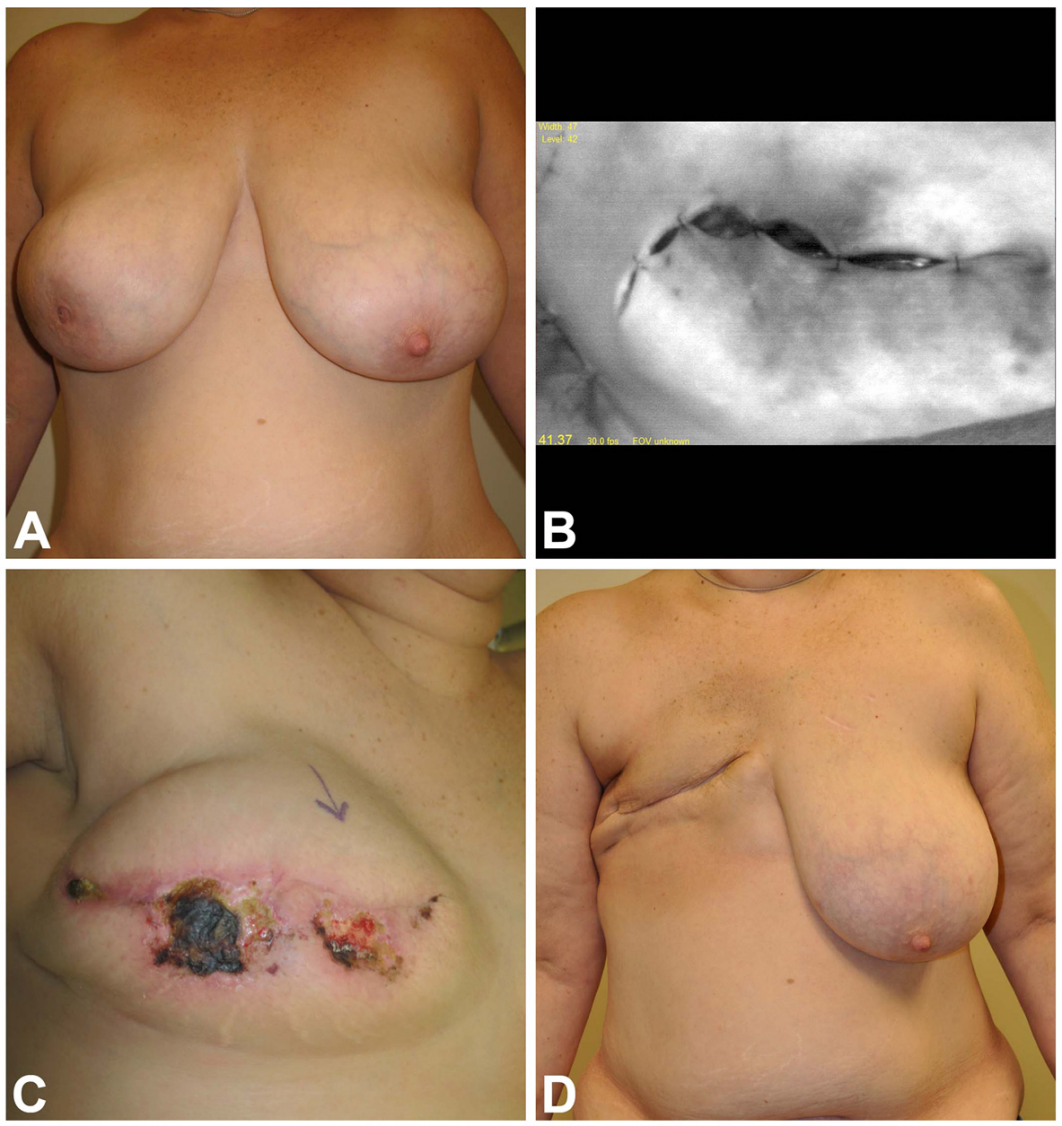
**In this case, a 45-year-old woman with right breast cancer (Panel A) requested expander insertion to maintain domain during chemotherapy and radiation therapy prior to autologous conversion. **SPY image (Panel **B**) identified poor perfusion in an extensive area around the incision. Clinically, the skin appeared well perfused and was preserved based on clinical judgment. Postoperative ischemia ensued, leading to full-thickness necrosis (Panel **C**). Two attempts at salvage with debridement and expander deflation failed to achieve successful closure, and the expander was removed to allow the patient to proceed with chemotherapy and radiation therapy. The resulting healed, radiated mastectomy site prior to free TRAM reconstruction is shown in Panel **D**.

**Figure 3:**
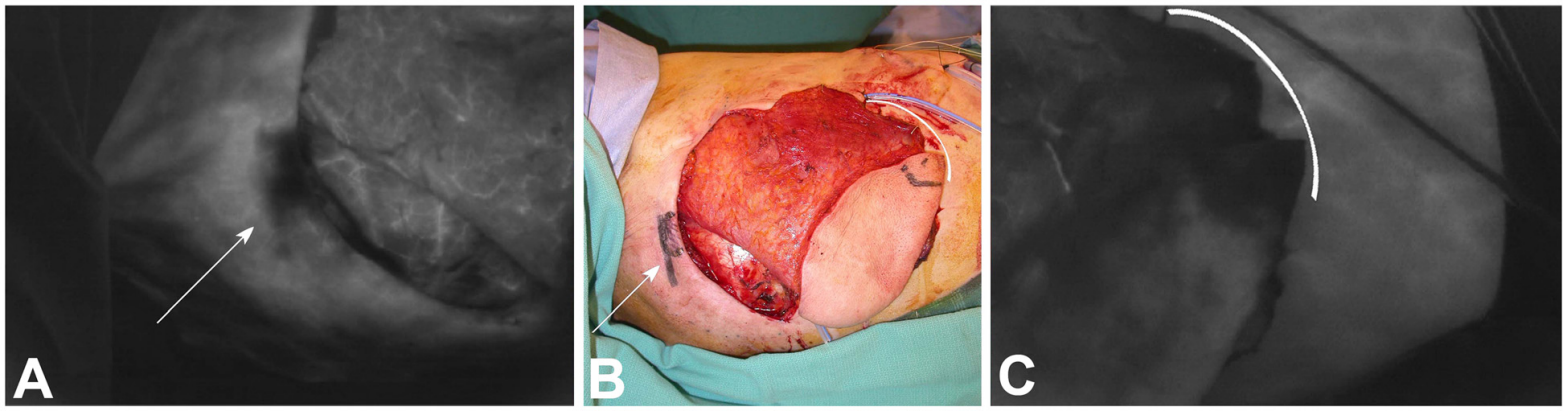
**Latissimus dorsi (LAT) flap evaluated using SPY. **Necrosis of the tips of transversely oriented LAT flaps can occur in some patients; the use of SPY identifies these regions intraoperatively. Panel **A **shows a SPY image of a LAT flap (head to the left, legs to the right) following rotation to the chest for breast reconstruction. The image shows two parallel scars from a previous biopsy with evidence of poor blood supply across the scars. This area was resected (arrow). Similarly, Panel **C **shows poor perfusion in the tip of the LAT flap and its underlying muscle (bracket). It is assumed that these regions of LAT flaps in some patients are outside the primary angiosome of the thoracodorsal artery and are supplied by smaller vessels that are divided in the normal harvest of the flap. In both cases, the tissues look perfectly normal clinically (Panel **B**, arrow and bracket). Each of these regions should be debrided prior to use in reconstruction. As illustrated by this case, variable anatomy can lead to areas of poor perfusion, despite good flap design and excellent surgical technique, that can only be identified through intraoperative imaging.

**Figure 4:**
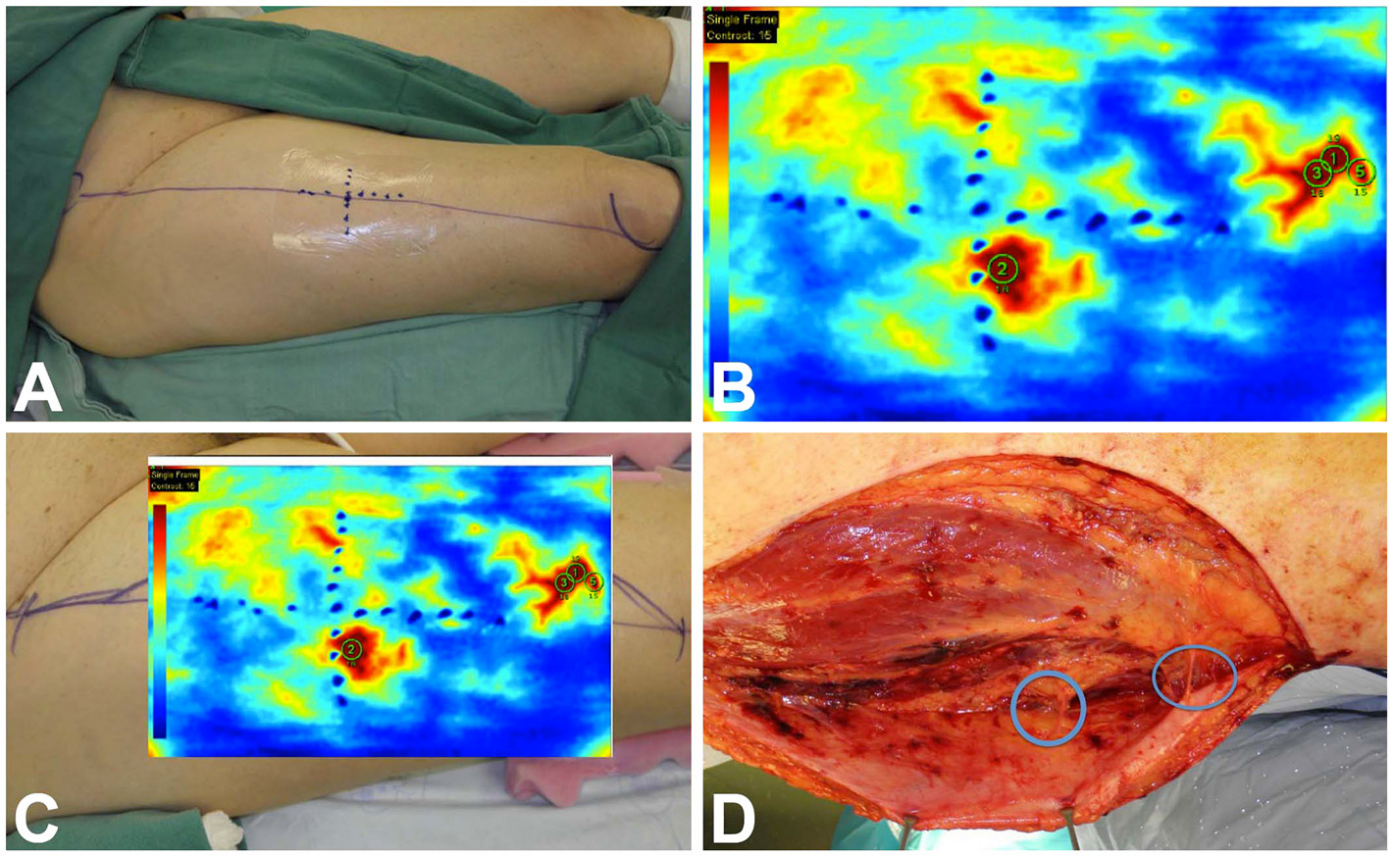
**Illustration of the use of SPY to optimize placement of skin paddle for harvest of the anterolateral thigh (ALT) flap for reconstructive surgery. **The proposed center of the ALT flap, in the right thigh, is marked in standard fashion at the midpoint of a line drawn between the anterior superior iliac spine (**A**) and the lateral patella (P) in preparation for harvest (Panel **A**). The colorized SPY Q analysis, shown in Panel **B**, identifies perforator perfusion zones in red along the AP line axis. The color SPY image is transposed over the native thigh in Panel **C**, showing the locations of perforator perfusion zones. Partial elevation of the ALT flap (Panel **D**) shows perforators (circled) emanating from the vastus lateralis muscle and rectus femoris muscle, corresponding to the perforator perfusion zones identified by SPY in Panels **B **and **C**. SPY will often show significant variation in perforator location, exposing the weakness of standard flap design.

Using clinical experience and published evidence, the authors developed recommendations for the intraoperative use of the SPY System to assess tissue perfusion in reconstructive surgical procedures. These recommendations are not intended to be prescriptive, but rather, to serve as general principles for the use of the SPY System in the intraoperative evaluation of tissues during reconstructive surgical procedures. The overall principles agreed on by the authors are listed by indication in Table 3. Specific technical recommendations regarding the use of the SPY System are also provided, including dosing of ICG and timing of evaluation (Table 4). A partial list of the types of flaps that may be appropriate for evaluation with SPY is provided in Table 5. The authors’ use of SPY in various reconstructive applications is illustrated in Figures 1, 2, 3 and 4.

**Table 3:** Overall recommendations regarding use of the SPY System in reconstructive procedures, by flap type

**Application**	**Points of use for ICG intraoperative laser angiography**
Free flaps	1. Identify perforator perfusion zone in donor site prior to incision; select optimal perforator and design flap
	2. Confirm flap perfusion during dissection, testing and comparing different perforators
	3. Confirm flap perfusion after transfer
	4. Confirm patency of arterial and venous anastomoses
	5. Detect areas of venous congestion by re-imaging 5–20 min after administration of ICG; can be performed following flap dissection, transfer, and/or inset
Pedicle flap	1. Identify perforator perfusion zone in donor site prior to incision; design flap
	2. Evaluate arterial and venous perfusion after elevation of flap and prior to transfer
	3. After transfer and after inset, confirm arterial inflow and venous return
Skin flap	1. Evaluate perfusion prior to incision; design flap
	2. After dissection, confirm flap perfusion
	3. After transfer and inset, confirm perfusion of flap
Mastectomy flap	1. Following mastectomy, confirm integrity of vascular perfusion in mastectomy flaps; select delayed vs. immediate reconstruction; select implant vs. expander reconstruction
	2. Confirm perfusion after insertion of reconstructive modality; determine expander volume or implant size; determine skin paddle size
	3. If revisions made, confirm perfusion in flaps

**Table 4:** Technical recommendations for use of the SPY System

**Application**	Timing of evaluation*
**Microvascular reconstructive surgery**	
Pre-incision identification of perforators	15-30 sec
Following dissection, confirmation of adequate flow and limits of perfusion	1-2 min
Following transfer, evaluation of arterial and venous anastomoses	Arterial phase: instantaneous.
	Venous phase: 30–60 sec
	Re-image at 2 min; if venous congestion is suspected, evaluate again at ≥4 min
Following inset, confirmation of adequate flow and limits of perfusion	1-2 min (Wait at least 10 min following previous ICG administration)
**Pedicle flap reconstruction**	
Pre-incision identification of perforator perfusion zone	≤1 min
Following elevation of flap, confirmation of adequate flow and limits of perfusion (selection of skin and soft tissue for preservation)	1-2 min
Following transposition and inset, confirmation of adequate flow and limits of perfusion	1-2 min
Skin flap reconstruction**	
Following elevation of skin flap, define limits of perfusion for flap design and detect sub-clinical ischemia	1-2 min
Following transposition and inset to confirm adequate perfusion	1-2 min (Wait at least 10 min following previous ICG administration)
**Mastectomy followed by TE/I reconstruction**	
Pre-mastectomy, map the vessels and the perfusion surrounding the nipple-areolar complex	15-30 sec
Following mastectomy, assess perfusion along the skin/tissue edges of the flap^†^ Wait 30–45 minutes following completion of mastectomy procedure to ensure recovery of perfusion. If no fluorescence is detected after this time period, additional wait time of up to 30 minutes may be appropriate.	3-4 min
With implant or tissue expander in place, evaluate mastectomy skin flap prior to filling expander	Wait 5 min after insertion of implant or tissue expander before imaging
After filling tissue expander, evaluate skin flaps, nipple-areolar complex, and surrounding tissue perfusion	Wait 5 min after filling tissue expander before imaging

*TE/I *= tissue expander/implant.

* Start recording after first appearance of fluorescent blush. Times given indicate when within the captured image sequence assessments should be made.

**For areas or conditions associated with reduced perfusion (eg, lower extremities, vasculopathy), longer wait times may be required before evaluation.

Notes: The Instructions for Use supplied with SPY note that the 25 mg ICG should be reconstituted in 10 cc of normal saline, giving a concentration of 2.5 mg/cc [15]. The Instructions for Use suggest administration of 10 mg (or 4 cc) ICG for visualization of tissue perfusion. For restudy, wait ≥10 min from previous injection of ICG. Take a baseline image before restudy to ensure that ICG has washed out. If complete wash out is needed, wait 15 minutes from previous ICG administration.

**Table 5:** Examples of flap types and procedures appropriate for imaging with ICG intraoperative laser angiography

Mastectomy and other skin flaps	Mastectomy flaps
	Cervicofacial flaps
	Facelift
	Cheek flap
	Cervical advancement flap
	Forehead flap
	Skin flap over pectoralis major
	Degloving injuries, upper and lower extremity
	Abdominal flaps (eg, hernia repair)
	Component separation
	Burn injuries
	Local flaps and adjacent tissue transfers:
	Bi-lobe flaps
	Propeller flaps
	Rhomboid flaps
Pedicle flaps	TRAM flap
	VRAM and extended VRAM flaps
	Deltopectoral flap
	Trapezius flap
	Myocutaneous pectoralis flap
	Latissimus dorsi flap
	Submental flap
	Supraclavicular artery flap
	Lateral intercostal artery flap
Free flaps	TRAM flap
	DIEP flap
	SIEA flap
	ALT flap
	Scapular/parascapular flaps
	Submental flap
	Fibular flap
	DCIA flap
	TUG/TMG flap
	GAP flap
	PAP flap
Breast reconstruction	Implant reconstructions
	Tissue expander reconstructions

#### Free flaps

The sequence of steps at which perfusion assessment with ICG for free flaps should be considered is illustrated in Table 3. The surgeon may consider performing the technique prior to any manipulation of the donor site to aid in flap design, during harvest of the flap to aid in vessel selection, and after flap elevation to aid in determining which parts of the flap should be used and where they should be positioned.

The pattern of ICG fluorescence can also be used to evaluate venous return. With SPY, venous congestion manifests as an area of brightness that persists for 5 to 20 minutes after administration of ICG. Persistent fluorescence often appears “hyper-white” on the monitor, suggesting that plasma-bound ICG is trapped within the perfusion zone. Re-imaging the flap 5 to 20 minutes after ICG injection can identify venous congestion following dissection, transfer, and/or inset. Persistent fluorescence in a free flap suggests the presence of venous thrombosis, a kinked vein, or improperly designed flap. With repeated injections of ICG (i.e., ≥4 injections), there may be some residual background fluorescence that must be distinguished from venous congestion.

#### Pedicle flaps

For pedicle flaps, the SPY System can be used to identify perforator perfusion zones and optimize design of the skin paddle over the perfusion zone (see Table 3). Once the flap has been designed and elevated, ICG may be administered to confirm viability of the flap prior to transfer and again following inset. After each of these steps, the ICG fluorescence pattern can help to identify problems, such as compressed or kinked vessels in the pedicle, as well as areas of poor perfusion that may require debridement or adjustments to flap inset, such as delay of inset, minimizing postoperative tissue necrosis.

#### Mastectomy flaps

Following completion of a mastectomy procedure, the SPY System can help to determine the viability of mastectomy flaps (see Table 3). This information can be used to maximize use of the mastectomy skin, thereby improving aesthetics by minimizing the need for larger skin paddles from transferred flaps and minimizing scar burden.

#### Implant reconstruction

The SPY System can provide information about the effects of tension and pressure on perfusion in the mastectomy flap, which may help to inform decisions regarding immediate implant placement or expander inflation volume. When tissue expanders are used, the effect of expander volume on skin flap perfusion can be monitored in real time, and decisions can be made regarding appropriate intraoperative fill volumes [1,13,68]. A final image may be obtained to confirm adequate flap perfusion following modification to the expander, implant, or skin.

#### Skin-sparing and nipple-sparing procedures

The SPY System can be used to evaluate perfusion of the mastectomy skin and the nipple-areolar complex (NAC) during a skin-sparing, nipple-sparing mastectomy. After completion of mastectomy, the NAC can be evaluated by examining both its superficial and deep surfaces to help determine whether the NAC should be retained. By examining the NAC with a sizer in place, the SPY System can aid in the decision to go straight to implant or to perform a staged reconstruction, minimizing risk of necrosis in the areas at risk.

#### Other skin flaps

Intraoperative laser angiography can be utilized to evaluate perfusion in multiple types of skin flaps (see Table 5 for examples). Imaging can be performed prior to incision to evaluate perfusion in the donor site and design the flap, during flap elevation to select the number and location of perforating vessels, following dissection to confirm viability of the flap, and again after transfer and after inset to confirm perfusion and rule out compression or kinking that may have occurred after flap transfer. Improved perfusion assessment can also assist in the decision to delay reconstruction, if perfusion is determined to be inadequate.

#### Benefits in challenging morphologies

In the authors’ experience, intraoperative laser angiography has particular clinical utility in cases where clinical judgment may be challenging, such as patients with darker skin tones and/or severe bruising or other tissue damage. Pigmentation or discoloration of the skin interfere with clinical assessment but are transparent to the SPY system. Perfusion across scars can also be visualized using SPY technology, whereas clinical assessment may be of limited utility.

#### Amount and timing of ICG administration and coordination with image capture

The recommendations include specific guidance regarding the method and timing of ICG administration, the timing of image capture with the SPY System, and the use of repeat evaluations during the same procedure (see Table 4). ICG is administered via intravenous bolus, using the best available venous access (central or peripheral), followed by a 10 cc bolus of normal saline.

Positioning the SPY device, injecting the ICG, and capturing the images requires coordination between surgeon and anesthesiologist to ensure that useful images are acquired. Surgeons should ensure that the SPY camera is on and recording before the first blush of fluorescence is seen. Newer versions of the SPY System include a 5-second buffer whenever the camera is on; therefore, images will be captured from 5 seconds prior to initiation of recording. The maximum capture time is 4.5 minutes, but additional scans may be performed immediately after this period, extending the study without additional injection of ICG [15].

#### Special considerations

Factors that may affect the use of the SPY System are listed in Table 6. Characteristics associated with ischemia (eg, smoking and peripheral vascular disease) may lead to reduced fluorescence and longer wait times following administration of ICG. Dyes, such as methylene blue, can interfere with the fluorescent image. Vasoconstrictors, such as epinephrine, drastically diminish blood flow and preclude accurate estimation of normal tissue perfusion.

**Table 6:** Variables that may impact use of modalities to evaluate tissue perfusion

Characteristics associated with ischemia	Previous radiation treatment
	Previous or aggressive surgery*
	Current smoking
	Obesity
	Diabetes
	Vasculopathy
	Chronic corticosteroid use
	Thin mastectomy flaps
Agents that interfere with imaging techniques	Methylene blue
	Lymphazurin blue
Agents that affect blood flow	Vasoconstrictors (eg, epinephrine)**

The SPY-Q Analysis Toolkit companion software allows for quantification of perfusion by assigning numeric values to intensity of fluorescence. Two types of values can be used: relative and absolute. Relative values are normalized to a reference value based on an image taken from tissue outside the area of interest, but within the field of view. Absolute values pertain strictly to the intensity of fluorescence signal in any given image. In the opinion of the authors, and based on the currently available technology, absolute values may have greater utility for quantification of perfusion than relative values, which depend on an arbitrary reference image from an area of normal perfusion. The utility of these values for prediction of necrosis remains to be determined. An analysis of images from a study of breast reconstruction using this software [12] suggested that quantification using absolute values may be useful to stratify tissue perfusion into three categories: viable tissue, tissue at high risk for necrosis, and tissue that should be evaluated in correlation to other clinical factors. Future studies may further clarify the roles for these values in prediction of postoperative complications.

Other factors that should be considered when using the SPY System include turning off ambient lighting to improve fluorescent image viewing (and on for visible photography), positioning the camera head perpendicular to the body surface being imaged, ensuring the patient is normothermic and normotensive, and administering ICG through the largest available intravenous access.

#### Safety of ICG

ICG has an excellent safety profile, with an adverse event rate of approximately 1 in 42,000 patients [69,70]. Anaphylactic reactions are rare, but can occur in patients with iodine sensitivity. The recommended maximum dose of ICG is 2 mg/kg [15].

### Summary

The elevation of tissue of any type results in potential devascularization and the risk for vascular compromise. The use of simple, reliable, and accurate adjunctive modalities to assist the surgeon in determining vascular compromise or poor perfusion will provide surgeons the opportunity to intervene early and minimize risk for postoperative complications.

The SPY Intraoperative Perfusion Assessment System has clinical utility for the perioperative visualization of tissue perfusion in multiple settings. The authors propose recommendations regarding the applications and most effective use of this technology for a range of plastic and reconstructive procedures. As this technology is more widely adopted and studied in the reconstructive setting, refinements to these technique recommendations may be made.

## Competing interests

Funding for the consensus development process was provided by LifeCell Corporation, Branchburg, NJ.

Dr. Geoffrey C. Gurtner is a consultant for LifeCell Corp.

Dr. Glyn E. Jones is a consultant for LifeCell Corp.

Dr. Peter C. Neligan has nothing to disclose.

Dr. Martin I. Newman is a consultant for LifeCell Corp.

Dr. Brett T. Phillips has nothing to disclose.

Dr. Justin M. Sacks is a consultant/speaker for LifeCell Corp.

Dr. Michael R. Zenn is a consultant for LifeCell Corp and Novadaq Technologies.

## Authors' contributions

All authors contributed to the conception of the work and participated in the design and review of the manuscript.

## Products discussed

SPY® Intraoperative Perfusion Assessment System (distributed in North America by LifeCell Corp., Branchburg, NJ; manufactured by Novadaq Technologies Inc., Richmond, British Columbia, Canada).
